# Omnivorous Behaviour of the Agouti (*Dasyprocta leporina*): A Neotropical Rodent with the Potential for Domestication

**DOI:** 10.1155/2019/3759783

**Published:** 2019-05-02

**Authors:** Kegan Romelle Jones, Kavita Ranjeeta Lall, Gary Wayne Garcia

**Affiliations:** ^1^The Department of Basic Veterinary Sciences (DBVS), School of Veterinary Medicine (SVM), Faculty of Medical Sciences (FMS), University of the West Indies (UWI), Mt. Hope, Trinidad and Tobago; ^2^The Open Tropical Forage-Animal Production Laboratory (OTF-APL), Department of Food Production (DFP), Faculty of Food and Agriculture (FFA), The University of the West Indies (UWI), St. Augustine, Trinidad and Tobago

## Abstract

The agouti is a Neotropical rodent which is mainly utilized for its meat in rural communities. Recently, captive rearing of these animals by wildlife farmers have increased in the Neotropics. This short communication consists of observation of feeding behaviour of captive reared agoutis at the University of the West Indies Field Station in Trinidad and Tobago. This is the first time in Trinidad and Tobago that meat consumption and the omnivorous behaviour of the agouti have been documented in the literature. The consumption of chicken (*Gallus domesticus*) eggs, dead chickens, and a brown dove (*Zenaida macroura*) by captive reared agoutis was noted. This document described the omnivorous behaviour of the agouti which is primarily considered a frugivorous animal. Similar studies in South America have shown that wild and captive reared agoutis consumed animal matter. Further work must be done on the dietary needs and nutrient requirements of the agouti at different physiological states.

## 1. Introduction

The agouti is a Neotropical rodent has been reported to inhabit South America, Central America, Mexico, and the Caribbean Antilles [1]. These animals have been hunted for their meat in rural areas. It was one of the several species described as mini livestock which can be utilized by rural communities as an essential source of meat protein [2]. The agouti has been reared in captivity and has potential for domestication [1]. The authors have stated that the agouti diet consisted of mainly fruits and seed [3]. Other authors categorized the agouti as frugivores and herbivores [4].

There is a dearth information on the biology of the agouti, but recently in the Caribbean and South America, some research work has been conducted. Recently, a study on the anatomy of the digestive system has been done, and it was identified that the small intestines of the agouti were larger in comparison to the rabbit. These animals also had a large caecum which facilitates hindgut fermentation [5]. The preferred feed particle size in feeding agoutis was found to be 12.7 mm × 25.4 mm for captive reared animals [6]. Gross anatomy of the male and female reproductive systems in the agouti was investigated in Trinidad; electroejaculation was performed in the male animal [7, 8]. In South America, analysis of blood hormones (oestrogen and progesterone) and the oestrus cycle was investigated in the agouti, and the duration of the cycle was approximately 28 days [9]. Pathogen harboured by the agouti was reviewed but no apparent clinical effects on the animals were noted [10]. Gastrointestinal parasitic organisms were found in wild agoutis in Trinidad, but no effect on the animals' health or performance was observed [11, 12]. Further work reviewed endoparasites of domesticated animals that originated from the Neotropics. These Neotropical animals had parasitic organisms present, but authors failed to report the effect but chose to only view these animals as reservoirs for diseases [13]. In captive reared animals, parasitic organisms have also been identified [14, 15], but no effects on the animals were reported either clinically or subclinically [16]. A parasite is defined as an organism which is present in the host and affects the host clinically or subclinically [17].

Recent works on blood parameters of agoutis have been reported, and there was no difference seen in blood reference values between male and female animals. However, there were differences in serum biochemical values among sexes [16, 18–21]. If diets are to be formulated to feed these animals in captivity, then reference blood values will be essential in identifying the effect that diets will have on the agouti's performance. In the literature, the agoutis have been classified as frugivores, herbivores, or opportunistic feeders with little attention given on this animal's omnivorous behaviour. The manuscript attempts to report a situation where captive reared agoutis consumed chicken (*Gallus domesticus*) eggs, a dead chicken, and a brown dove (*Zenaida macroura*). Thus, the omnivorous behaviour of these animals which were historically given little attention is highlighted.

## 2. Methodology

### 2.1. Location and Climate

The University of the West Indies (UWI) Field Station is located at Mt. Hope, Trinidad (10°38′16″N and 61°25′41″W). Trinidad and Tobago is located in the humid tropics with an average temperature of 32°C to 22°C, rainfall of 1000 mm, and humidity of 82%.

### 2.2. Housing of Agoutis at University Field Station (UFS)

At the location where the observations on the agoutis were made, animals were housed in cages and on floor pens. At that location, there were over one hundred agoutis at all physiological states being reared. The floor pens population consisted of both sexes, and the female to male ratio was 5 : 1. In the cages, animals are reared as breeding pairs (male and female), maternity (pregnant female), and juveniles (less than 3 months of age).

### 2.3. Diets of Agoutis at the University Field Station

The diet of the agoutis consisted of local feed resources such as mangoes (*Mangifera indica*), papaya (*Carica papaya*), green bananas (*Musa* spp.), pumpkin (*Cucurbita pepo*), guavas (*Psidium guajava*), breadfruit (*Articarpus altilis*), and dasheen (*Colocasia esculenta*). The agoutis' diet was also supplemented with Rabbit Ration® (17% CP) and chicken (*Gallus domesticus*) eggs.

## 3. Results

### 3.1. Observations of Captive Reared Agoutis

On 14^th^ October 2018 at 9:00 am before the animals in the floor pens were fed and cleaned, the observer noticed a live brown dove (*Zenaida macroura*) enter the pen. The observer entered the floor pen and took photographic evidence of the consumption of the brown dove by the adult female agouti. The dove landed in the water trough and was unable to fly because of its soaked feathers. The immobilized dove was then killed by an adult female.

**Figure 1:**
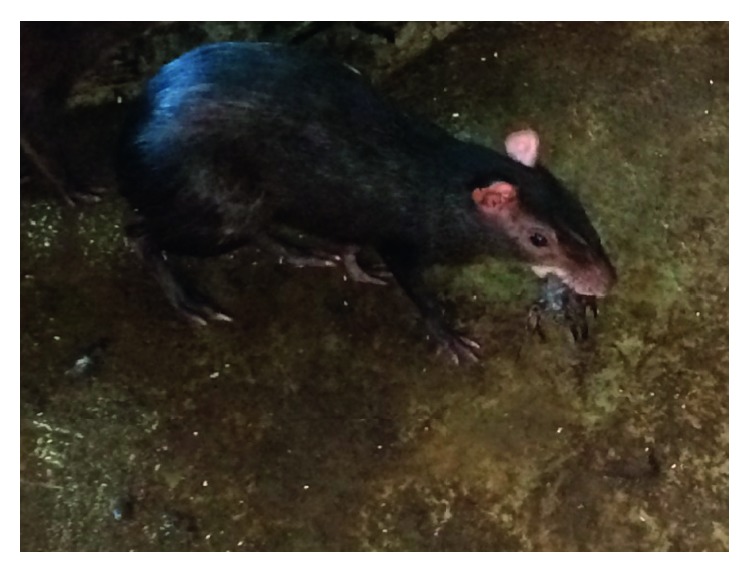
Female agouti holding dove (*Zenaida macroura*) in mouth before consumption at the University Field Station.

**Figure 2:**
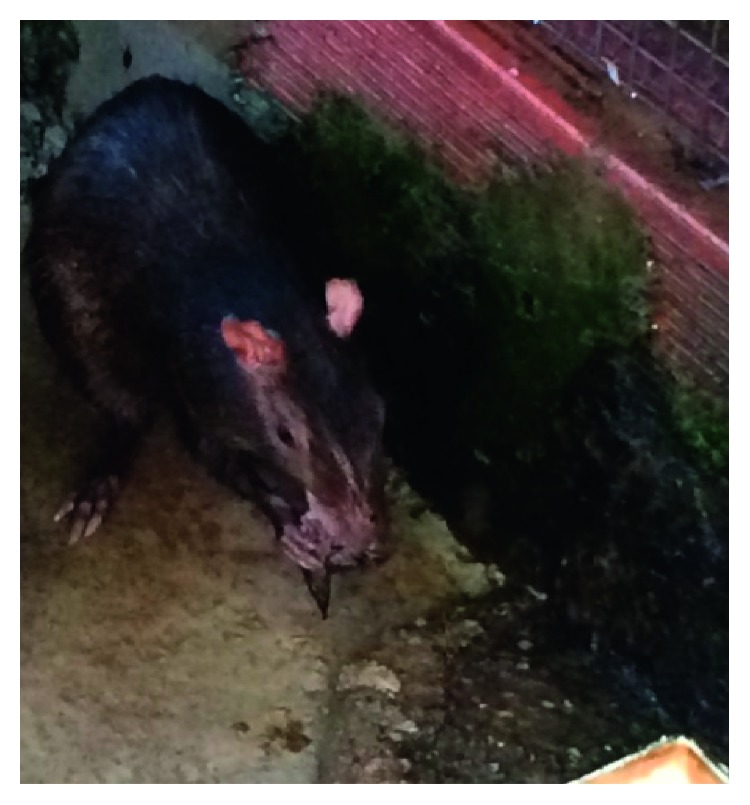
Female agouti consuming dove in a sitting position while manipulating carcass with forelimbs.

Frank blood was seen on the floor of the pen where the dove was killed. The dove was held in the animal's mouth before the consumption of the carcass began (Figure 1). The female then proceeded to consume the dove using its forelimb to hold the carcass. To consume the carcass, the animal adopted the position similar to the consumption of fruits and seed (Figure 2). The animal ate the entire carcass including head and limbs, and the feathers of the bird remained on the floor. The consumption began at 9:10 am, and the carcass was consumed by 9:55 am.

**Figure 3:**
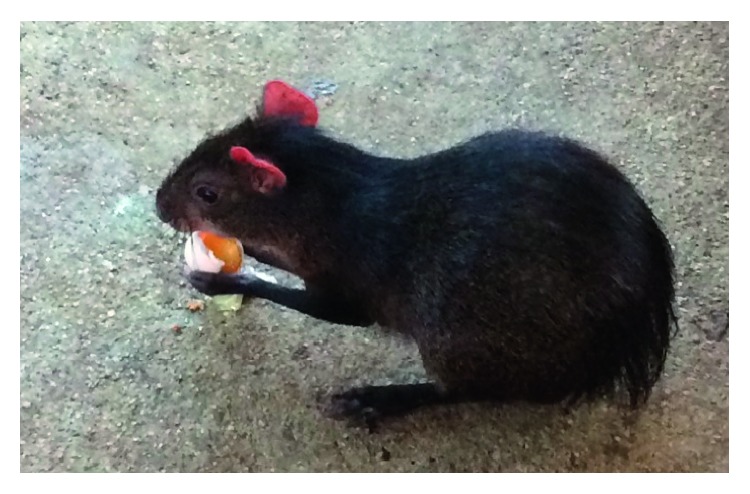
Agouti consuming chicken (*Gallus domesticus*) egg while assuming a sitting position.

**Figure 4:**
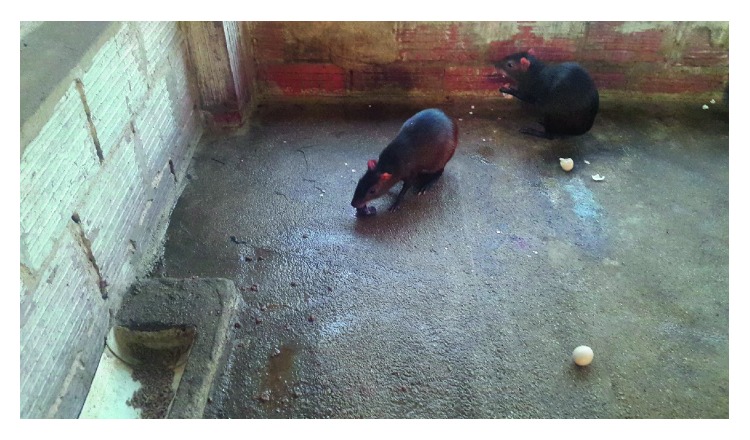
Agoutis consuming eggs with foetus inside. Animals consumed foetus, egg content, and shells.

The consumption of chicken eggs was similar to the consumption of fruits. The animal held the egg with its forelimbs. The eggs were cracked using their sharp incisors, and the egg shell and contents were consumed by the agouti (Figure 3). On a later date, the agoutis were fed fertile eggs and the agoutis ate the foetus, egg contents, and egg shells (Figure 4). All animals present in the unit at UFS were captive reared with an average age of six (6) years.

## 4. Discussion

This short communication showed that the Agouti (*Dasyprocta leporina*) consumed brown doves and chicken eggs (animal matter). It is the first time that agoutis in Trinidad have been reported to have consumed animal matter. Similar reports have been recorded in captivity where an agouti killed and consumed a male *Liomys pictus* [22]. The authors have described the agouti as a frugivore with major components of the diet comprising fruit and seeds [22]. However, animals in the order *Rodentia* have been described as omnivores with the majority of the animals in this order consuming fruits, seeds, animal matter, leaves, and fibre [23]. Omnivorous behaviour has been described for other members of the order *Rodentia*, but no records were made on *Dasyprocta* spp. [23]. The dentitions of members of the order *Rodentia* were described as having three functions which were cropping, insectivorousness, and flesh eating [23].

In Brazil, *Dasyprocta leporina* in the wild was reported to have consumed bruchid larvae found in palm nuts [24]. Further works reported that stomach contents of wild *Dasyprocta leporina* in French Guiana had 16.4% animal matter [25]. There was no significant difference in the animal matter found in the stomach of the agouti at different times of the year [25]. Investigators in the French Guiana forest found agoutis' stomach containing an average of 6.2% animal matter (insects) [26].

In Tijuca National Park, a reintroduced female *D. leporina* was found eating carrion of a tapiti (*Sylvilagus brasiliensis*) [27]. The agouti ate the carrion in a similar manner to what was reported in this study. The carcass was held in the forelimbs, and the animal sat on in the hind limbs during consumption. The consumption of chicken eggs by the agouti in this report was also recorded in Brazil. Brazilian agoutis (*Dasyprocta leporina* and *Dasyprocta azarae*) which were reared in captivity with chickens (*Gallas domesticus*) and geese (*Anas anas*) consumed the eggs of both poultry species [28].

In Brazil, workers investigated the predatory behaviour of the agouti (*Dasyprocta azarae*). An experiment was set up using two groups of animals: the first group consisted of animals caught from the wild, while the second group was born in captivity. In both groups, immediate predation of eggs and chicks was noticed [29]. The results of the experiment in Brazil showed that the zoophagic behaviour in the agouti was innate which was similar to what was observed in this study. The animals present in this study were never given chicken (*Gallas domesticus*) eggs and were all born in captivity but when eggs were offered they consumed it immediately. Some authors in the review of the literature stated these animals were frugivores with a capacity to be omnivorous due to their dental structure [30].

At this time, there is a dearth of information in the literature on the nutrient requirement of the agouti. In the Guianan forest, the lappes' (*Agouti paca*) diet contained more fruit, seed, and leaves than the agouti. However, the agouti consumed more insects than the lappe [26]. A preferential study on dietary items consumed by the lappe revealed that items with highest energy content were consumed more readily than feed items with lower energy content [31]. However, the study did not offer the lappe any animal matter but only fruits and vegetables. In Costa Rica, using teeth marking and faecal sampling, it was found that the lappe utilized approximately thirty-three (33) species of trees. However, the investigation did not identify the consumption of animal matter by the lappe [32]. The agouti and the lappe have been described as frugivores that consumed fruits, leaves, and seeds in their diets [33]. Similar work was done in Brazil, and the lappe was found to consume twelve species of trees belonging to ten families and ten genera [34].

Capybaras (*Hydrochoerus hydrochaeris*) reared in cultivated areas in Brazil consumed rice, corn, sugar cane, water hyacinth, and *Brachiaria* sp. [35]. A similar study was done on a biological reserve in Brazil, and the capybara was found to have consumed 133 species of herbaceous vegetation using faecal analysis and vegetation collection techniques [36]. A study in Argentina revealed that these animals selected plants based on their caloric energy content [36]. With respect to Neotropical rodents with the potential for domestication, the agouti (*Dasyprocta leporina*) has omnivorous feeding habits when compared to the lappe (*Agouti paca*/*Cuniculus paca*) which was found to be a strict frugivore and the capybara (*Hydrochaerus hydrochaeris*) was a strict herbivore.

## 5. Conclusions

Observation on the agoutis born in captivity in Trinidad and Tobago showed that they consumed a brown dove (*Zenaida macroura*), a dead chicken carcass, and chicken (*Gallus domesticus*) eggs. These findings were in agreement with previous observations in Brazilian literature which noted the zoophagic behaviour of the agouti (*Dasyprocta leporina*) in the wild and in captivity. However, this is the first time that this behaviour has been recorded in Trinidad and highlights the fact that these animals which were considered to be frugivores were actually omnivorous in feeding behaviour.

## 6. Future Directions

If agoutis (*Dasyprocta leporina*) are to be reared in captivity for production, then the balanced diet of the animal must be known. Feeding trials must be conducted to evaluate the effect of diets containing different levels of the animal material (insects, meat, and eggs), fruits, seeds, and leaves to understand which composition is best for each physiological state.

## Data Availability

The data used to support the findings of this study are included within the article.
